# Enhanced reticulospinal output in patients with (*REEP1*) hereditary spastic paraplegia type 31

**DOI:** 10.1007/s00415-013-7178-6

**Published:** 2013-11-13

**Authors:** K. M. Fisher, P. F. Chinnery, S. N. Baker, M. R. Baker

**Affiliations:** 1 Institute of Neuroscience, The Medical School, Newcastle University, Framlington Place, Newcastle upon Tyne, NE2 4HH UK; 2 Mitochondrial Research Group, Institute of Genetic Medicine, International Centre for Life, Newcastle University, Central Parkway, Newcastle upon Tyne, NE1 3BZ UK; 3 Department of Neurology, Royal Victoria Infirmary, Queen Victoria Road, Newcastle upon Tyne, NE1 4LP UK; 4 Department of Clinical Neurophysiology, Royal Victoria Infirmary, Queen Victoria Road, Newcastle upon Tyne, NE1 4LP UK

Dear Sirs,

The striking paradox of pure autosomal dominant hereditary spastic paraplegia (AD HSP), in contrast to capsular stroke or primary lateral sclerosis, for example, is that despite extensive corticospinal tract (CST) degeneration and prominent lower limb spasticity, leg weakness is not a prominent early feature of the disease. Therefore, other descending motor pathways presumably compensate for CST degeneration, though, until the recent article by Nonnekes et al. [1] in the *Journal of Neurology*, this assumption had remained unproven. Using startling acoustic stimuli (SAS), they showed that the reticulospinal tract (RST) is not only functioning in patients with HSP, but that it compensates for lower limb deficits of postural control caused by CST degeneration.

In most patients with pure AD HSP, electrophysiological evidence of CST disease is limited to the lumbosacral cord [2]. However, some AD HSP genotypes (e.g., *SPG4*) are associated with a more severe phenotype and have motor-evoked potential (MEP) abnormalities in the upper limbs [3–5], consistent with post-mortem evidence of CST degeneration at all levels, from the medulla to the lumbo-sacral cord [6].

We tested MEPs (see Supplementary Methods) in two patients (father and son) with *SPG31* AD HSP (*REEP1* exon 5 c.337C > T/p.Arg113X [7]). In patient 2 (age 42; disease duration 37 years) MEPs were ‘typical’ of pure AD HSP [8], with prolonged central motor conduction times (CMCTs) in the lower limbs (CMCT 22.2 ms) and normal CMCTs in the upper limbs. However, in patient 1 (age 68; disease duration 64 years) CMCTs were significantly prolonged in the upper limbs (Fig. 1).

**Fig. 1:**
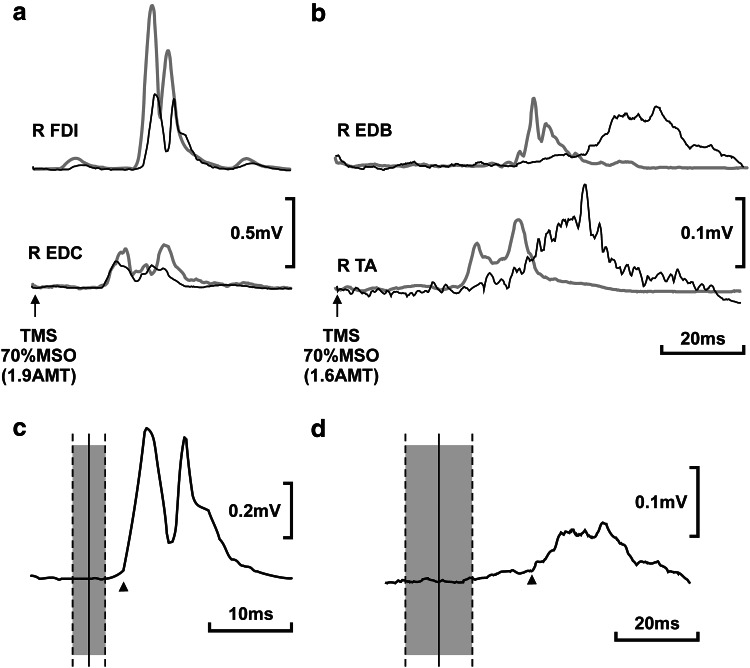
Examples of rectified motor-evoked potentials (MEPs) obtained from a 68-year-old patient with SPG31/*REEP1* HSP (*black*) and an age-matched control (*grey*), aligned to the stimulus. **a** Upper limb MEPs recorded from right *first dorsal interosseous* (R FDI) and *extensor digitorum* (R EDC) muscles and evoked with a single transcranial magnetic stimulus (indicated by an *arrow*) at 70 % of maximum stimulator output (MSO), equivalent to 1.9 times the active motor threshold (AMT; for details see Supplementary Methods). Each trace is an average of 20 individual MEPs. Voltage calibration *bars* apply to both patient and control MEPs. **b** Lower limb MEPs recorded from right *extensor digitorum brevis* (R EDB) and *tibialis anterior* (R TA) muscles using a stimulus strength of 1.6x AMT (70 % MSO). **c** Right upper limb (FDI) central motor conduction time (CMCT). **d** Right lower limb (EDB) CMCT. In **c** and **d**, peripheral motor conduction times were subtracted from the FDI and EDB MEPs shown in **a** and **b**, revealing the CMCT and the result plotted on a longer time base. Solid *vertical lines* show the mean CMCT within the normal population (aligned to the start), and the *grey* boxes between *vertical* *dashed lines* extend two standard deviations above and below the mean [14]. *Arrow* heads indicate MEP onset

Given the absence of clinical upper limb weakness in patient 1, we measured EMG onset latencies in a Start-React paradigm to see whether the RST might be compensating for the CST deficit in the upper limbs [9–11] (Supplementary Methods), as illustrated in Fig. 2a. Experiments had the relevant institutional ethical approval and complied with the Declaration of Helsinki, and were performed in both patients and 11 controls, aged 56–82 years. Traditionally, the effects of SAS on the visual reaction time (VRT), and the visual start-react time (VSRT), are normalized, thus:

1 $$ \Updelta {\text{VRT}}\left( \% \right) = \frac{{\left( {\text{VRT - VSRT}} \right)}}{\text{VRT}} \times 100. $$

**Fig. 2:**
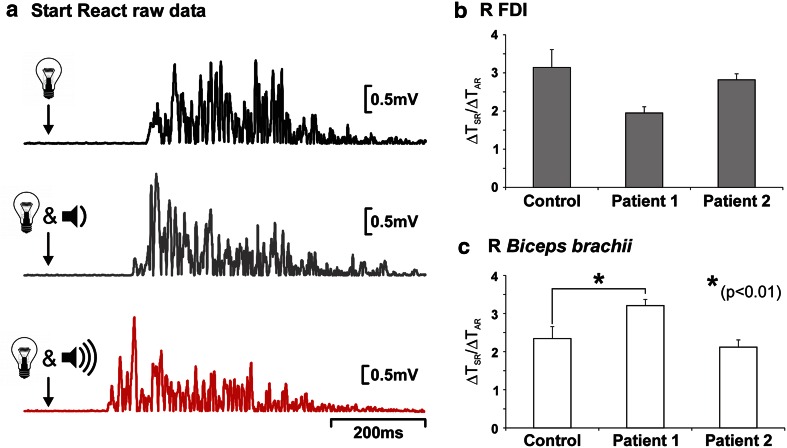
**a** Example of normal raw rectified EMG data recorded from right *biceps brachii* in a 61-year-old male control subject performing the Start-React task. The EMG burst from which the VRT was measured is plotted in *black*, that from which the ART was derived is plotted in *grey* and the VSRT response is plotted in *red*. **b**, **c** Mean ∆*T*_SR_ */*∆*T*_AR_ ratios calculated from EMG onset latencies measured in Start-React experiments are plotted. **b** Right *first dorsal interosseous* (R FDI) and **c** right biceps brachii (R BB). Error bars are standard error of the mean. Note that in patient 1, the R BB ratio is significantly increased (one-sample *t* test; *p* < 0.01)

The normal ∆VRT is ~50 % [9], and a ∆VRT of less than 50 % is indicative of disease affecting the RST. However, because we were interested in measuring any change in the gain of the RST output, accessed via auditory pathways, we have used a ratio that incorporates the auditory reaction time (ART) following a low-intensity sound, as follows:

2 $$ \Updelta T_{\text{SR}} /\Updelta T_{\text{AR}} = \frac{{ ( {\text{VRT }} - {\text{VSRT)}}}}{{ ( {\text{VRT}} - {\text{ART)}}}} $$

where ∆*T*_SR_ is the shortening effect of a SAS on the visual reaction time and ∆*T*_AR_ measures the shortening of reaction time provided by a non-startling auditory stimulus, which presumably does not activate RST pathways. The results of this analysis are shown in Fig. 2c, d. Patient 2, who had no evidence of cervical CST disease, had normal ∆*T*_SR_/∆*T*_AR_ ratios. However, patient 1, who had MEP evidence of cervical CST disease, had significantly increased ratios, but only when measured from *biceps brachii* EMG, despite normal ARTs and VRTs (Supplementary Results/Fig. 2). This result supports the notion that the RST mitigates the effects of disease within the cervical CST. The RST appears to compensate by increasing its output gain by a factor of around 1.5. Although the RST does project to both proximal and distal upper limb muscles [12], the effects of SAS are only seen in distal muscles in some tasks [13], possibly explaining why we detected differences only in the biceps muscle. These observations suggest that therapeutic interventions aimed at increasing the gain of RST outputs could improve recovery from neurological disorders characterized by CST dysfunction.

## Electronic supplementary material

Below is the link to the electronic supplementary material.

Supplementary material 1 (DOCX 242 kb)

## References

[1] Nonnekes J, de Niet M, Nijhuis LB, de Bot ST, van de Warrenburg BP, Bloem BR, Geurts AC, Weerdesteyn V (2013). Mechanisms of postural instability in hereditary spastic paraplegia. J Neurol.

[2] Lang N, Optenhoefel T, Deuschl G, Klebe S (2011). Axonal integrity of corticospinal projections to the upper limbs in patients with pure hereditary spastic paraplegia. Clin Neurophysiol.

[3] Bonsch D, Schwindt A, Navratil P, Palm D, Neumann C, Klimpe S, Schickel J, Hazan J, Weiller C, Deufel T (2003). Motor system abnormalities in hereditary spastic paraparesis type 4 (SPG4) depend on the type of mutation in the spastin gene. J Neurol Neurosurg Psychiatry.

[4] Schady W, Dick JP, Sheard A, Crampton S (1991). Central motor conduction studies in hereditary spastic paraplegia. J Neurol Neurosurg Psychiatry.

[5] Pelosi L, Lanzillo B, Perretti A, Santoro L, Blumhardt L, Caruso G (1991). Motor and somatosensory evoked potentials in hereditary spastic paraplegia. J Neurol Neurosurg Psychiatry.

[6] Deluca GC, Ebers GC, Esiri MM (2004). The extent of axonal loss in the long tracts in hereditary spastic paraplegia. Neuropathol Appl Neurobiol.

[7] Hewamadduma C, McDermott C, Kirby J, Grierson A, Panayi M, Dalton A, Rajabally Y, Shaw P (2009). New pedigrees and novel mutation expand the phenotype of REEP1-associated hereditary spastic paraplegia (HSP). Neurogenetics.

[8] Polo JM, Calleja J, Combarros O, Berciano J (1993). Hereditary “pure” spastic paraplegia: a study of nine families. J Neurol Neurosurg Psychiatry.

[9] Valldeoriola F, Valls-Sole J, Tolosa E, Ventura PJ, Nobbe FA, Marti MJ (1998). Effects of a startling acoustic stimulus on reaction time in different parkinsonian syndromes. Neurology.

[10] Valls-Sole J, Kumru H, Kofler M (2008). Interaction between startle and voluntary reactions in humans. Exp Brain Res.

[11] Rothwell JC (2005) The Startle reflex, voluntary movement, and the reticuopsinal tract. In: Cruccu G, Hallett M (eds) Brainstem Function and Dysfunction (Supplement to Clinical Neurophysiology), (Elsevier) volume 58, pp. 221–229

[12] Davidson AG, Buford JA (2006). Bilateral actions of the reticulospinal tract on arm and shoulder muscles in the monkey: stimulus triggered averaging. Exp Brain Res.

[13] Honeycutt CF, Kharouta M, Perreault EJ (2013). Evidence for reticulospinal contributions to coordinated finger movements in humans. J Neurophysiol.

[14] Eisen AA, Shtybel W (1990). AAEM minimonograph #35: clinical experience with transcranial magnetic stimulation. Muscle Nerve.

