# Supplementary material for: Enhanced reticulospinal output in patients with (REEP1) hereditary spastic paraplegia type 31
Source: J Neurol. 2013 Nov 13;260(12):3182–4. doi: 10.1007/s00415-013-7178-6 (PMC3843367; doi:10.1007/s00415-013-7178-6)
Supplement: Supplementary file 1 — Supplementary material 1 (DOCX 242 kb) [file 415_2013_7178_MOESM1_ESM.docx]

# ENHANCED RETICULOSPINAL OUTPUT IN PATIENTS WITH (*REEP1*) HEREDITARY SPASTIC PARAPLEGIA TYPE 31

### KM Fisher, PF Chinnery, SN Baker, MR Baker

### *Journal of Neurology*

### Corresponding Author

Dr Mark R Baker, Institute of Neuroscience, The Medical School, Newcastle University,
Framlington Place, Newcastle upon Tyne, NE2 4HH, UK
E-mail: [m.r.baker@ncl.ac.uk](mailto:m.r.baker@ncl.ac.uk)

## Supplementary Methods

### Electromyogram (EMG) recordings

Surface EMG was recorded with disposable adhesive electrodes (Biosense Medical Ltd, UK) placed over *first dorsal interosseous* (FDI), *extensor digitorum communis* (EDC), *flexor digitorum superficialis* (FDS), *biceps brachii* (BB), *extensor digitorum brevis* (EDB) and *tibialis anterior* (TA), *medial gastrocnemius* (MG). Electrodes were positioned with a separation of approximately 20mm, such that the non-inverting contact was over the motor point and the reference electrode was over the tendon. Surface EMG Signals were bandpass filtered at 30Hz-2KHz before being digitized at 5KHZ by a 1401 (CED, UK) interface, connected to a PC running Spike 2 (CED, UK) software.

### Motor evoked potentials (MEPs)

Motor cortex was stimulated using a Magstim 200 stimulator (The Magstim Co Ltd, Whitland, UK). A 13cm outside diameter circular transcranial magnetic stimulation (TMS) coil was used for obtaining upper limb MEPs, with current direction optimised for activation of each hemisphere (A side up: left hemisphere; B side up: right hemisphere). Lower limb MEPs were obtained using a double cone TMS coil (anterior coil current: right hemisphere; posterior coil current: left hemisphere). Coils were initially placed over the vertex and then moved in small increments to optimise MEPs; stimuli were delivered at 0.2Hz.

MEP threshold over motor cortex was determined whilst subjects provided a gentle background contraction. Active motor threshold (AMT) was defined as the stimulus intensity at which MEPs of at least 0.05 mV could be observed in 50% of stimulus-triggered EMG traces at rest or 0.2mV in active muscles [[1](#_ENREF_1), [2](#_ENREF_2)]. MEPs were recorded from FDI, EDC and FDS in the upper limb and EDB, TA and MG in the lower limb using adhesive electrodes while subjects maintained a background muscle contraction of ~5% maximum voluntary contraction (MVC). TMS output intensity was set at 2xAMT or the highest level tolerated by the patient (and at least 20% above AMT) to ensure accurate measurement of central motor conduction time [[3](#_ENREF_3)]. If the threshold was >80% maximum stimulator output (MSO) then the stimulator was set to 100% (or the highest level tolerated by the patient). Central motor conduction time (CMCT) was calculated by subtracting the peripheral motor conduction time (PMCT) from the MEP latency. PMCT was calculated using the F and M response latencies as follows:

$$PMCT=\frac{(F+M-1)}{2}$$

Published normative data [[4](#_ENREF_4)] was used to assess CMCTs. A MEP was considered to be abnormal if the CMCT was increased by more than two standard deviations compared to normative data.

### StartReact experiments

The Start-React paradigm used in these experiments was modified from standard published procedures [[5](#_ENREF_5), [6](#_ENREF_6)]. Surface EMG was recorded from right FDI and BB. Subjects (and patients) were seated comfortably and asked to observe a red light-emitting diode (LED) located approximately 1m in front of them. When it was illuminated, they were required to immediately perform an upper limb flexion task; specifically, this comprised contraction of the right biceps muscle whilst making a fist with the hand. The LED was illuminated for 50ms. The latency from the stimulus to the onset of the EMG burst in BB (or FDI) was defined as the visual reaction time (VRT). The VRT is typically 180-200 ms [[7](#_ENREF_7)]. On some trials, the visual stimulus was co-presented with either a quiet acoustic stimulus (<95dB; 50ms) or a startling acoustic stimulus (SAS; >115dB; 50ms). The time delay between co-presentation of the quiet acoustic stimulus to the onset of the EMG response was termed the auditory reaction time (ART), which is normally 140-160 ms [[7](#_ENREF_7)], and the delay between the SAS and EMG onset defined the visual+startle reaction time (VSRT). Subjects/patients were excluded if they could not perform the task or if there was evidence of hearing impairment preventing measurement of the ART.

At the start of the recording, there were 5 consecutive SAS; these were presented in order to habituate the startle. Patients were not required to respond to these stimuli. Subsequently, stimuli were randomized and presented with intervals of 5-10 seconds. Approximately 20 responses were recorded for each condition.

The effects of SAS in the Start-React paradigm are mediated via the RST [[8](#_ENREF_8)]. In order to compare the effects of startling stimuli in patients and age-matched controls we used a novel normalisation procedure, which also allowed us to estimate changes in the gain of reticulospinal outputs. Whilst both the ART and VSRT are mediated via the cochlear and vestibular nuclei, only VSRT activates the RST and thus the gain of the RST output can be estimated by:

$$\frac{(VRT - VSRT)}{(VRT-ART)}\text{=}\frac{\Delta T_{SR}}{\Delta T_{AR}}\text{ }\text{ }$$

Demographics and raw numerical data obtained from 11 age-matched control subjects are tabulated below for FDI (Supplementary Table 1) and BB (Supplementary Table 2).

Data quality was inspected by eye before analysis in MATLAB (Mathworks, Natick, MA, USA). To investigate the effect of age on *ΔT_SR_*/*ΔT_AR_* we divided the control groups into two subgroups; a group of 6 aged less than 58 years and a group of 5 aged 58 years or more. We found no statistical difference between these groups and therefore for the purposes of investigating changes in RST output gain in HSP patients we treated this dataset as one. Moreover, although most controls were female, we found no statistical difference between male and female *ΔT_SR_*/*ΔT_AR_* ratios.

## Supplementary Results

The results of Start-React experiments in 2 patients with *REEP1* HSP are shown in Supplementary Table 3.

The VRT and ART are thought to be mediated via the corticospinal tract whereas the VSRT is mediated by the reticulospinal tract. Potentially therefore, if the CST is severely diseased and the VRT and ART consequently pathologically prolonged, the ∆T_SR_/∆T_AR_ ratio could be significantly increased without a change in RST gain (i.e. VSRT). To be certain therefore that any increase in the ∆T_SR_/∆T_AR_ ratio was attributable to an increase in RST gain, we compared ART, VRT and VSRT between healthy controls and patients. The results of this analysis are shown in Supplementary Figure 2. There was no statistical difference between these measures, suggesting that CST pathways in patient 1, whilst abnormal on MEP testing, did not affect the VRT or ART, and therefore that the increase in the ∆T_SR_/∆T_AR_ ratio in Patient 1 represent an adaptive increase in RST output gain.

Supplementary Table 1. Control data for the Start-React paradigm, including *ΔT_SR_/ΔT_AR_* ratios, measured from *first dorsal interosseous* EMG onset latency.

| **ID** | **Age** | **Sex** | **VRT (ms)** | **VSRT (ms)** | **ART (ms)** | **VRT-VSRT (ms)** | **VRT-ART (ms)** | ***ΔT_SR_/ΔT_AR_*** |
| --- | --- | --- | --- | --- | --- | --- | --- | --- |
| 3CF03 | 53 | male | 214.11 | 137.68 | 183.99 | 76.43 | 30.12 | 2.537517 |
| 2PS02 | 48 | female | 206.6 | 115.01 | 153.42 | 91.59 | 53.18 | 1.722264 |
| 1WC06 | 82 | female | 286.86 | 199.27 | 236.52 | 87.59 | 50.34 | 1.739968 |
| 1ZF08 | 58 | female | 261.01 | 208.78 | 249.35 | 52.23 | 11.66 | 4.479417 |
| 2MF06 | 57 | female | 304.19 | 184.22 | 262.65 | 119.97 | 41.54 | 2.88806 |
| 1KL02 | 63 | male | 234.54 | 143.3 | 209.18 | 91.24 | 25.36 | 3.597792 |
| 1BP02 | 61 | male | 203.44 | 146.29 | 176.83 | 57.15 | 26.61 | 2.147689 |
| 3HR02 | 57 | female | 258.56 | 179.58 | 247.24 | 78.98 | 11.32 | 6.977032 |
| 1LS02 | 46 | female | 252.23 | 198.01 | 238.31 | 54.22 | 13.92 | 3.895115 |
| 1JK05 | 54 | female | 270.38 | 172.88 | 234.14 | 97.5 | 36.24 | 2.690397 |
| 2EH02 | 63 | female | 290.3 | 242.32 | 264.54 | 47.98 | 25.76 | 1.862578 |

Supplementary Table 2. Control data for the Start-React paradigm, including *ΔT_SR_/ΔT_AR_* ratios, measured from *biceps brachii* EMG onset latency.

| **ID** | **Age** | **Sex** | **VRT (ms)** | **VSRT (ms)** | **ART (ms)** | **VRT-VSRT (ms)** | **VRT-ART (ms)** | ***ΔT_SR_/ΔT_AR_*** |
| --- | --- | --- | --- | --- | --- | --- | --- | --- |
| 3CF03 | 53 | male | 197.32 | 141.41 | 165.95 | 55.91 | 31.37 | 1.782276 |
| 2PS02 | 48 | female | 205.78 | 115.15 | 139.87 | 90.63 | 65.91 | 1.375057 |
| 1WC06 | 82 | female | 219.85 | 136.32 | 174.32 | 83.53 | 45.53 | 1.834615 |
| 1ZF08 | 58 | female | 191.02 | 129.5 | 161.83 | 61.52 | 29.19 | 2.107571 |
| 2MF06 | 57 | female | 306.52 | 177.33 | 211.37 | 129.19 | 95.15 | 1.357751 |
| 1KL02 | 63 | male | 201.75 | 113.01 | 181.54 | 88.74 | 20.21 | 4.390896 |
| 1BP02 | 61 | male | 181.06 | 125.37 | 167.3 | 55.69 | 13.76 | 4.047238 |
| 3HR02 | 57 | female | 185.6 | 123.84 | 159.85 | 61.76 | 25.75 | 2.398447 |
| 1LS02 | 46 | female | 227.27 | 172.96 | 207.79 | 54.31 | 19.48 | 2.787988 |
| 1JK05 | 54 | female | 201.26 | 128.49 | 162.86 | 72.77 | 38.4 | 1.895052 |
| 2EH02 | 63 | female | 250.85 | 202.97 | 224.07 | 47.88 | 26.78 | 1.787901 |

Supplementary Table 3. Average Start-React data obtained from both male patients and measured from *first dorsal interosseous* and *biceps brachii* EMG onset latencies.

| **ID** | **Age** | **Muscle** | **VRT (ms)** | **VSRT (ms)** | **ART (ms)** | **VRT-VSRT (ms)** | **VRT-ART (ms)** | ***ΔT_SR_/ΔT_AR_*** |
| --- | --- | --- | --- | --- | --- | --- | --- | --- |
| Patient 1 | 68 | FDI | 236.55 | 154.4 | 194.38 | 82.15 | 42.17 | 1.948067346 |
|  |  | BB | 220.62 | 138.94 | 195.16 | 81.68 | 25.46 | 3.208169678 |
| Patient 2 | 42 | FDI | 245.95 | 177.13 | 221.53 | 68.82 | 24.42 | 2.818181818 |
|  |  | BB | 228.21 | 161.99 | 196.96 | 66.22 | 31.25 | 2.11904 |

Supplementary Figure 1. Comparison of mean *ΔT_SR_/ΔT_AR_* ratios calculated from first *dorsal interosseous* (FDI) and *biceps brachii* (BB) EMG data by age for controls <58 years of age (unfilled bars; n=6) and controls ≥58 years of age (grey bars; n=5). Error bars are standard error of the mean. There was no statistical difference at the 5% level between *ΔT_SR_/ΔT_AR_* ratios.


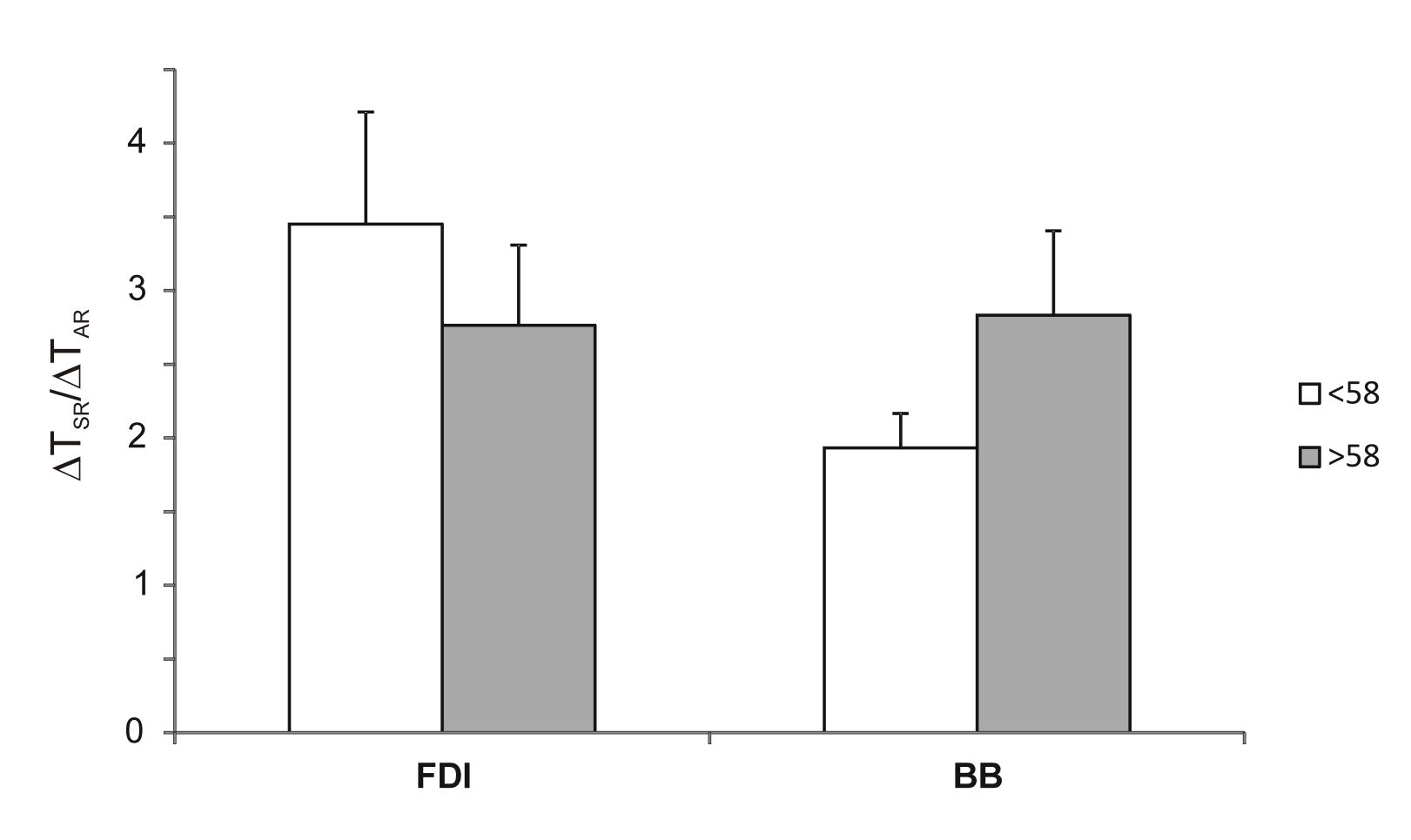


Supplementary Figure 2. Comparison of mean Visual reaction time (VRT), Auditory Reaction Time (ART) and Visual Start-React Time (VSRT) in controls (n=11) *REEP1* patients. ***A.*** Mean VRT, ART and VSRT measured from the onset of the rectified *biceps brachii* EMG in response to the stimulus. ***B.*** Mean VRT, ART and VSRT measured from the onset of rectified EMG recorded from right first *dorsal interosseous* (FDI). Error bars show standard deviations. There was no statistical difference at the 10% level between VRT, ART or VSRT in patients and controls (2-tail t test).


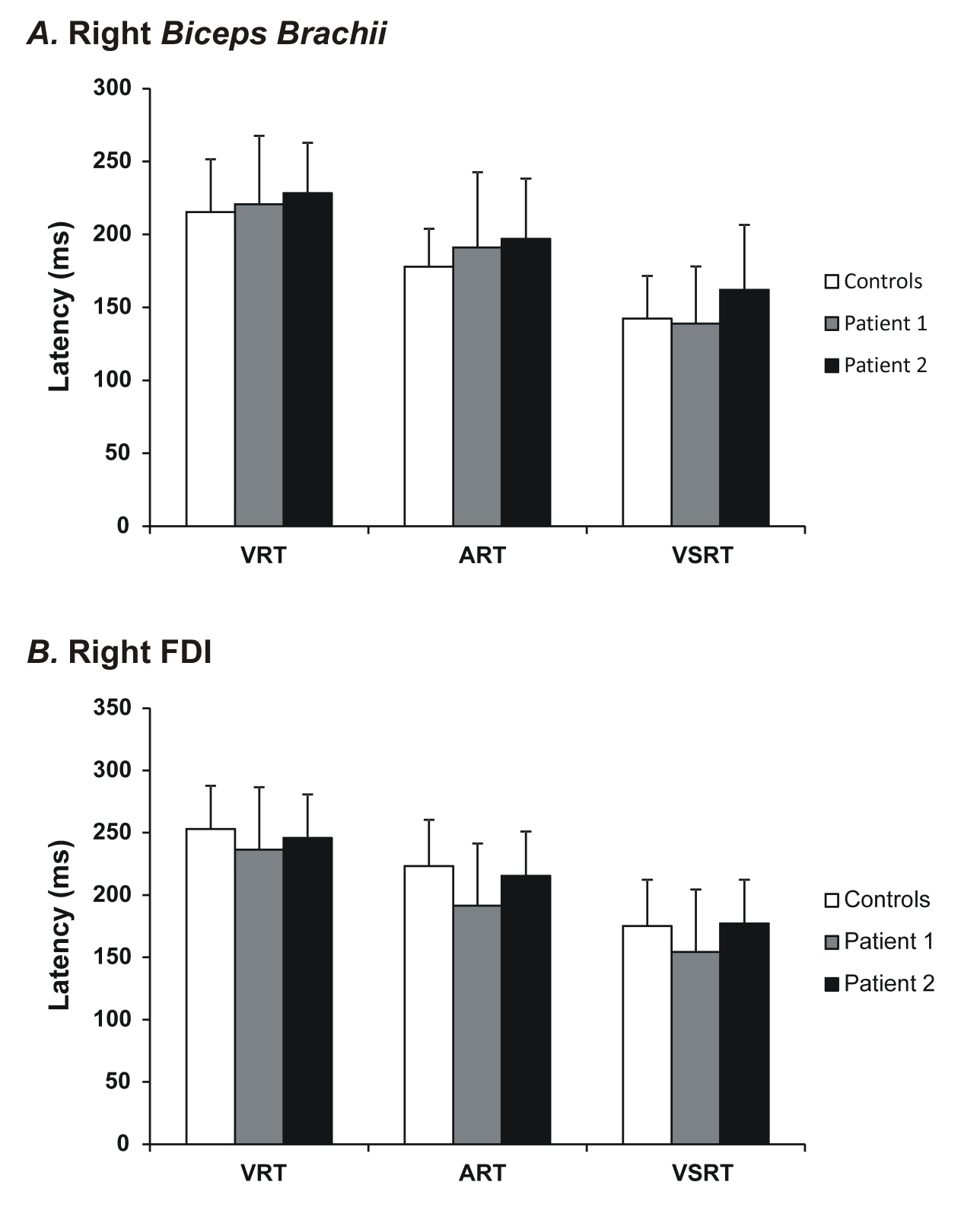


## Supplementary References

1. Chen, R., Cros, D., Curra, A., Di Lazzaro, V., Lefaucheur, J.P., Magistris, M.R., Mills, K., Rosler, K.M., Triggs, W.J., Ugawa, Y., et al. (2008). The clinical diagnostic utility of transcranial magnetic stimulation: report of an IFCN committee. Clinical Neurophysiology 119:504-532.

2. Hanajima, R., Wang, R., Nakatani-Enomoto, S., Hamada, M., Terao, Y., Furubayashi, T., Okabe, S., Inomata-Terada, S., Yugeta, A., Rothwell, J.C., et al. (2007). Comparison of different methods for estimating motor threshold with transcranial magnetic stimulation. Clinical Neurophysiology 118:2120-2122.

3. Hess, C.W., Mills, K.R., and Murray, N.M. (1987). Responses in small hand muscles from magnetic stimulation of the human brain. The Journal of Physiology 388:397-419.

4. Eisen, A.A., and Shtybel, W. (1990). AAEM minimonograph #35: Clinical experience with transcranial magnetic stimulation. Muscle & Nerve 13:995-1011.

5. Valldeoriola, F., Valls-Sole, J., Tolosa, E., Ventura, P.J., Nobbe, F.A., and Marti, M.J. (1998). Effects of a startling acoustic stimulus on reaction time in different parkinsonian syndromes. Neurology 51:1315-1320.

6. Valls-Sole, J., Compta, Y., Costa, J., Valldeoriola, F., and Rumia, J. (2008). Human central nervous system circuits examined through the electrodes implanted for deep brain stimulation. Clinical Neurophysiology 119:1219-1231.

7. Thompson, P.D., Colebatch, J.G., Brown, P., Rothwell, J.C., Day, B.L., Obeso, J.A., and Marsden, C.D. (1992). Voluntary stimulus-sensitive jerks and jumps mimicking myoclonus or pathological startle syndromes. Movement Disorders 7:257-262.

8. Rothwell, J.C. (2005). The Startle reflex, voluntary movement, and the reticuopsinal tract. In Brainstem Function and Dysfunction (Supplement to Clinical Neurophysiology), Volume 58, G. Cruccu and M. Hallett, eds. (Elsevier), pp. 221-229.
